# Supplementary material for: Multisecond ligand dissociation dynamics from atomistic simulations
Source: Nat Commun. 2020 Jun 10;11:2918. doi: 10.1038/s41467-020-16655-1 (PMC7286908; doi:10.1038/s41467-020-16655-1)
Supplement: Supplementary file 1 — Supplementary Information [file 41467_2020_16655_MOESM1_ESM.pdf]

Supplementary Information for

Multisecond ligand dissociation dynamics from atomistic simulations

Steffen Wolf,[a] Benjamin Lickert, Simon Bray, and Gerhard Stock[a]

[a]email: steffen.wolf@physik.uni-freiburg.de; stock@physik.uni-freiburg.de

SUPPLEMENTARY METHODS

MD simulation details.

Protein and ion interactions were described by the Amber99SB* force field[1,2], water molecules with the TIP3P model[3]. Simulations were carried out using Gromacs v2018 (Ref. 4) in a CPU/GPU hybrid implementation. Protein protonation states were evaluated with propka[5]. Van der Waals interactions were calculated with a cut-off of 1 nm, electrostatic interactions using the particle mesh Ewald method[6] with a minimal real-space cut-off of 1 nm. All covalent bonds with hydrogen atoms were constrained using LINCS[7]. After an initial steepest descent minimisation with positional restraints on protein and ligand heavy atoms, an initial 0.1 ns equilibration MD simulation in the NPT ensemble was performed with a 1 fs time step and positional restraints of protein and ligand heavy atoms. A temperature of 290.15 K was kept constant using the Bussi (v-rescale) thermostat[8] (coupling time constant of 0.2 ps), the pressure was kept constant at 1 bar using the Berendsen barostat[9] (coupling time constant of 0.5 ps), followed by a second steepest descent minimisation without restraints and a short 0.1 ns equilibration MD simulation in the NPT ensemble.

dcTMD calculations[10] were carried out using the PULL code implemented in Gromacs using the "constraint" option employing a SHAKE implementation[11]. 200–400 statistically independent start points of simulations were obtained by generating different atomic velocity distributions after the 10 ns unbiased simulations, all corresponding to a temperature of 290.15 K. After a 0.1 ns preequilibration using parameters as described above with positional restraints on protein and ligand heavy atoms and a constant distance constraint of all simulation systems, constant velocity calculations were carried out with $v_c = 1$ m/s covering a distance of 2 nm, switching the barostat to the Parrinello-Rahman barostat[12]. The constraint pseudo-force f_c was written out each time step.

NaCl. Free energy $\Delta G(x)$ and friction profiles $\Gamma(x)$ were obtained from 1000 trajectories of previous dcTMD calculations[10] at pulling velocity $v_c = 1$ m/s. For a better sampling, we continued the unbiased fully atomistic simulations described in Ref. 10 and extended them to a full microsecond of simulated time. As these simulations used a cubic simulation box, binding and unbinding waiting times cannot directly be compared to the results of our Langevin simulation with "reflective" borders (see below), which represent radial dynamics. To obtain data sets that allow such a comparison, we removed all time steps with $x < 0.265$ nm and $x > 1.265$ nm from MD trajectories, and calculated mean waiting times for the resulting cut $x(t)$ trajectories.

Trypsin-benzamidine. Benzamidine parameters were obtained using Antechamber[13] and Acpype[14] with atomic parameters derived from GAFF parameters[15]. Atomic charges were obtained as RESP charges[16] based on QM calculations at the HF/6-31G* level using Orca[17] and Multiwfn[18]. Trypsin (PDB ID 3PTB)[19] was placed into a dodecahedral box with dimensions of 7.5 x 7.5 x 5.3 nm^3 side length and solvated with 8971 water molecules. 16 sodium and 25 chloride ions were added to yield a charge neutral box with a salt concentration of 0.1 M[20]. After the initial equilibration, we added an additional 10 ns unbiased MD simulation to yield a converged protein structure. As pulling coordinates, we used the

distance between the center of mass of all benzamidine heavy atoms and the one of the C_α atoms of the central β-sheet of trypsin.

Hsp90-inhibitor. Parameters of the resorcinol inhibitor were taken from Ref. 21: here, inhibitor parameters were generated using Antechamber[13] and Acpype[14] with atomic parameters derived from GAFF parameters[15] and AM1-BCC atomic charges[22,23]. Solvated simulation boxes of the Hsp90-inhibitor complex were taken from Ref. 21 (compound **1j**), which in turn are based on the 2.5 Å X-ray crystal structure with PDB ID 6FCJ[24]. As in the case of trypsin, the distance between the center of mass of all ligand heavy atoms and the one of the C_α atoms of the central β-sheet of Hsp90 served as as pulling coordinate as used in Ref. 21.

Data evaluation. Minimal distance evaluation for contact determination was performed using the MDanalysis Python library[25], nonequilibrium principal component analysis was carried out using the fastpca program[26]. Data evaluation was carried out using a Jupyter notebook[27] employing the numpy[28], scipy[29] and astropy[30] Python libraries. Graphs were plotted using the matplotlib[31] Python library, molecular structures were displayed via PyMOL[32].

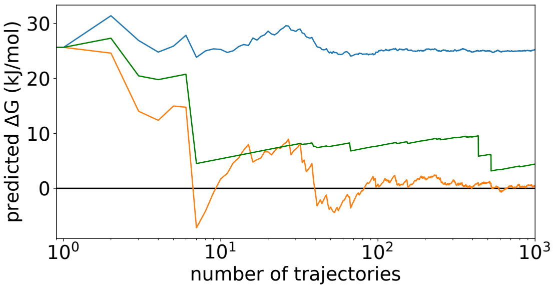

SUPPLEMENTARY FIGURE 1. **Convergence of dcTMD free energy estimate,** illustrated for a Gaussian work distribution. Compared are $\langle W \rangle$ in blue, ΔG estimate by Jarzynski's identity in green, cumulant expansion estimate in orange.

Statistical convergence of dcTMD free energy and friction estimates

To illustrate the statistical convergence of various quantities (such as mean work and free energy) calculated via the second-order cumulant expansion of Jarzynski's identity, we have performed a detailed study for a large range of ensemble sizes and pulling velocities in the case of NaCl (see the Supplementary Information of Ref. 10). Using a simple model problem, here we restrict us to demonstrate the clear improvement of the convergence behavior when the second-order cumulant expansion instead of the direct evaluation of Jarzynski's identity is used. To this end, we generated test data in form of draws from a normal distribution with mean $\langle W \rangle$ and variance $\langle \delta W^2 \rangle$ chosen such that $\Delta G = \langle W \rangle - \frac{1}{2k_\mathrm{B}T} \langle \delta W^2 \rangle = 0$. As Supplementary Figure 1 shows, the estimator from Jarzynski's identity exhibits a slow, erratic convergence behavior. On the other hand, the cumulant expansion-based estimator gives the dissipated work in terms of the variance of the nonequilibrium work, which is much easier to compute and converges to a fluctuation around $1k_\mathrm{B}T \approx 2.5$ kJ/mol after ~ 100 draws.

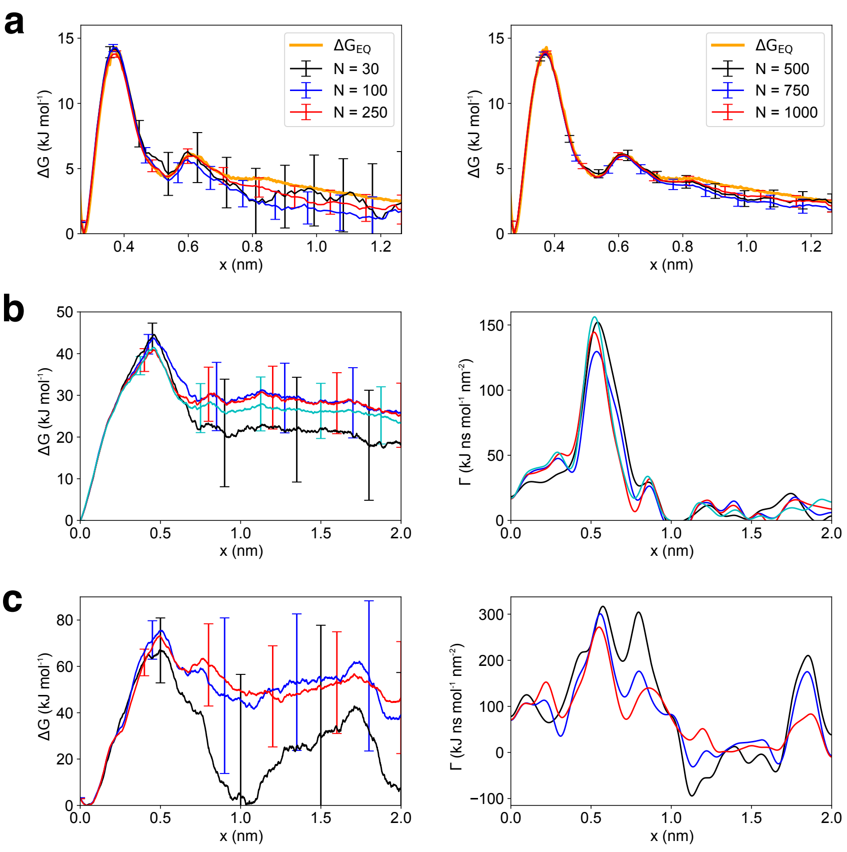

SUPPLEMENTARY FIGURE 2. **Jackknife analysis of free energy profiles and Gauss-smoothed friction (σ = 40 ps),** as obtained for **a** NaCl-water, **b** trypsin-benzamidine, and **c** Hsp90-inhibitor complex. Error bars denote the Jackknife standard error obtained for various numbers of TMD trajectories ("samples"). Color code in **b**: 52 samples in black, 84 in blue, 117 in red, 148 in cyan. Color code in **c**: 30 in black, 50 in blue, 93 in red.

In a similar vein, we analyzed the convergence of free energies and friction profiles in our three molecular simulation systems. To estimate an error of the free energy profiles, we resort to a Jackknife ("leave-one-out") analysis[33], which represents a superior method if we do not know the distribution that the free energy estimate follows. Because the estimate of the free energy depends on the estimate of both mean and variance of the nonequilibrium work (see the Theory section in the main text), the error of the free energy will decrease with the error of the 2nd moment of the nonequilibrium work, which is known to converge slowly[34]. Consequentially, while we expect the estimate of the mean free energy to converge comparatively fast, the error will converge slower.

Figure 2 displays the convergence of free energies and friction profiles of NaCl-water and the two investigated protein-ligand systems. In the case of the NaCl system, the free energy is within 1 $k_{\mathrm{B}}T$ of the free energy from unbiased simulations directly for N=30 simulations, while the error decreases to below 1 $k_{\mathrm{B}}T$ within N=100 simulations. Please see the Supplementary Information of Ref. 10 for the convergence of NaCl-water friction factors. For the trypsin-benzamidine system, free energies appear to converge with $\sim$100 trajectories, despite an error of $\sim$4 $k_{\mathrm{B}}T$. In a similar vein, the free energy estimates for the

Hsp90-inhibitor complex change for $\sim$2 $k_\mathrm{B}T$ after $\sim$100 trajectories. A similar convergence appears to apply to friction fields. We therefore expect about 100 trajectories per path to be sufficient to obtain a sufficiently converged free energy surface and friction profile for rate prediction.

Furthermore, the binding free energies of $\sim$ 27 kJ/mol (Trypsin) and $\sim$ 45 kJ/mol (Hsp90), calculated as the difference between ΔG at $x = 0$ nm and $x = 2$ nm, compare well to the standard free energies of binding $\Delta G^0 = 28.0$ kJ/mol (Ref. 20) and 40.7 ± 0.2 kJ/mol (Ref. 35), respectively, based on the experimentally measured $K_D = C_0 \exp\left(-\Delta G^0/k_\mathrm{B}T\right)$ with the standard reference concentration $C_0 = 1$ mol/l. We are aware that a distance-based free energy difference as used here and a standard free energy of binding can differ by several $k_\mathrm{B}T$ (see Ref. 36). We therefore base the goodness of our predictions in the main text on protein-ligand complex rates and the resulting K_D, respectively.

Lastly, we note that the convergence is best and errors are smallest directly around the main barrier. Therefore, predictions of unbinding rates will be more accurate than predictions of binding rates, despite being the slower and thus actually harder rate to predict.

Pathway separation

In the case of trypsin-benzamidine, pathway separation was performed by employing nonequilibrium principal component analysis[37] (PCA) using a covariance matrix based on protein-ligand contact distances[38]. To this end, the PCA included all minimal amino acid-ligand distances that are found below a cut-off distance of 4.5 Å in any snapshot of all pulling trajectories. Trajectories were projected onto the first two principal components and sorted according to pathways by visual inspection as displayed in Supplementary Figure 3. dcTMD calculations of free energy and friction were then carried out separately for such bundles of trajectories. Performing 200 pulling simulations of trypsin-benzamidine, we found 84 trajectories to constitute the major unbinding pathway (”middle” pathway in Supplementary Figure 3), for which free energy and friction profiles were converged, and whose free energy difference between bound and unbound state qualitatively agree with the standard free energy of binding known from experiment (see Supplementary Figure 2).

For the Hsp90-inhibitor complex, we employed a path separation based on geometric distances between individual trajectories[39]. After aligning trajectories with the protein's C_α atoms as fit reference, we calculated the matrix of means over time of the root mean square distance of ligand heavy atoms between individual trajectories. We then applied the NeighborNet algorithm[40] to the matrix to cluster trajectories according to distances. From the considered 400 trajectories, the cluster that gave a free energy difference between $x = 0$ nm and $x = 2$ nm that was closest to the experimental ΔG^0 calculated from the respecitve K_D[35] was taken by 93 single trajectories.

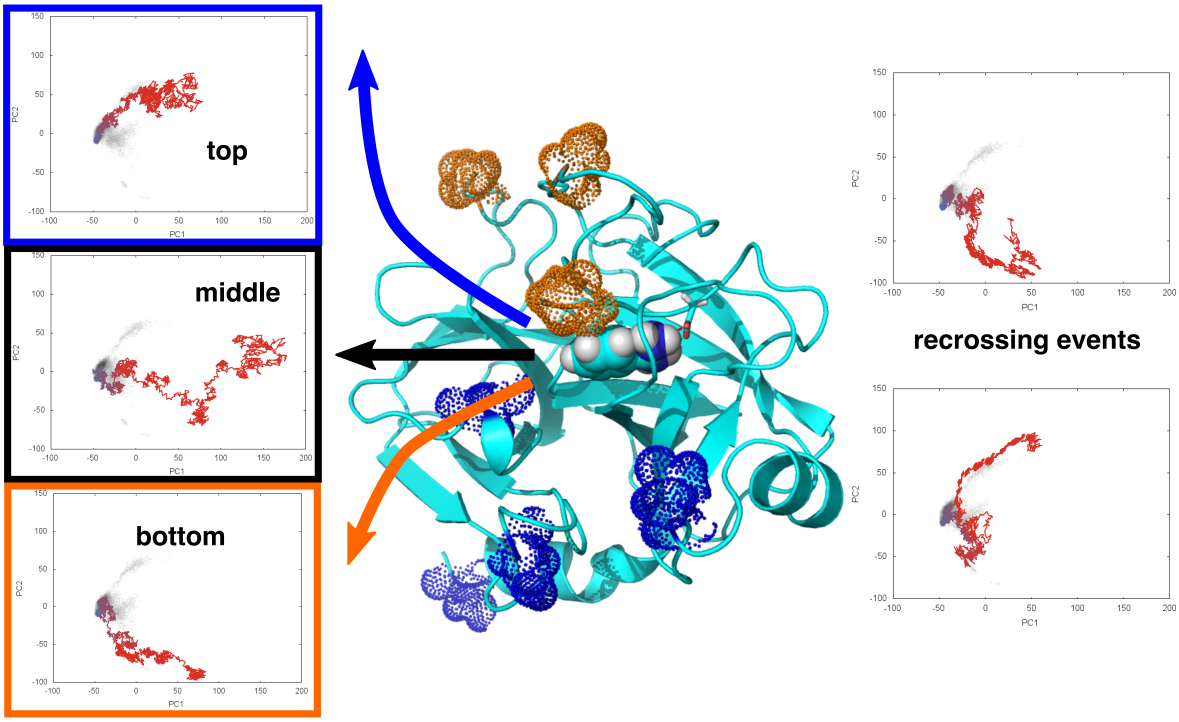

SUPPLEMENTARY FIGURE 3. **Pathways predicted by dcTMD for the unbinding of trypsin-benzamidine,** using a pulling velocity of $v_c = 1$ m/s. (Middle) Structure of trypsin (PDB ID 3PTB)[19] as cartoon, benzamidine as van der Waals spheres, Asp189 in sticks, residues characterizing PC2 as blue and orange dots, respectively. Arrows indicate the direction of pathways. (Left) Nonequilibrium PCA results display minima in nonequilibrium energies as grey shades, corresponding to temporary binding sites during unbinding. Projected single representative trajectories as lines are colored from blue to red according to time evolution. All trajectories start from a central minimum at (-50,0). Three main pathways (top, middle, bottom) can be observed: the "top" pathway passes through the elongated minimum at (0,50), the "middle" pathway through the central minimum around (-25,0), and the "bottom" pathway through a shallow minimum at ca. (-10,-70). The "middle" pathway is majorly populated. (Right) Additionally, trajectories can "recross" between the pathways: in the presented examples, trajectories first follow the "middle" pathway before crossing over to the "bottom" pathway at PC1 $\approx$ -20 nm (top) or jump between all three pathways at once (bottom).

Optimal pulling velocities

While the results presented above display the evaluation of single pathways after a successful pathway separation, being able to do so depends on finding a suitable pulling velocity v_c that allows to distinguish pathways. To obtain a nonequilibrium work profile that contains the least dissipative work and at the same time uses the most likely pathway between states, it intuitively appears to be the best choice to pull as slowly as possible. Supplementary Figure 4 displays nonequilibrium energy landscapes[37] obtained from dcTMD simulations of the trypsin-benzamidine complex, using 100 trajectories at v_c = 0.1, 1 and 10 m/s. We recognise a tradeoff between the pulling velocity and the structural resolution of the associated energy landscape. For 10 m/s, we hardly observe any structure in the first two PCs. Though this velocity is suited for a scoring of ligands according to unbinding kinetics[21], obviously a pathway separation cannot be performed. For 1 m/s, we observe several in-

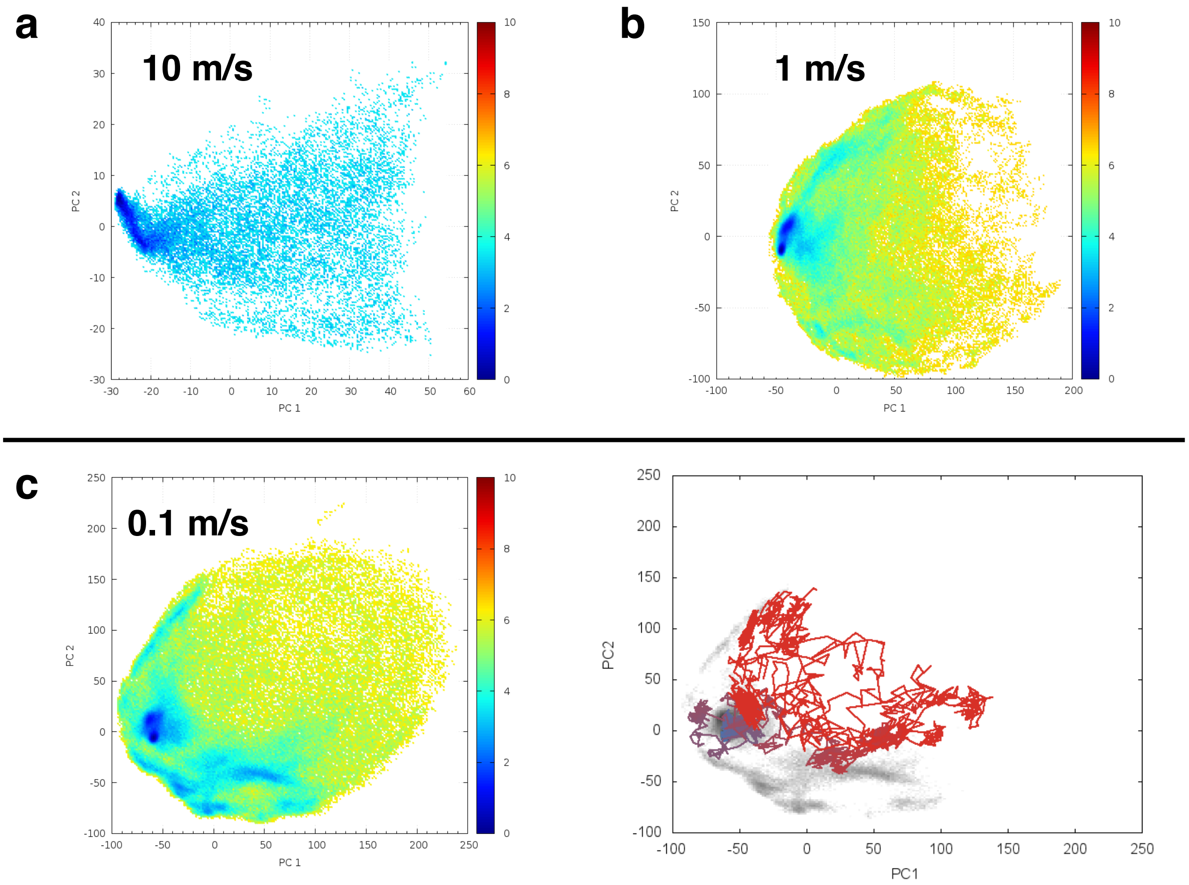

SUPPLEMENTARY FIGURE 4. **Velocity dependence of pathways in dcTMD simulations.** Nonequilibrium energy landscapes $\Delta\mathcal{G}(PC1, PC2) = -k_{\mathrm{B}}TP(PC1, PC2)$ obtained from dcTMD simulations of the trypsin-benzamidine complex, where $P(PC1, PC2)$ is the probability distribution of all dcTMD simulations projected on the first two principal components of a contact distance PCA[37]. For v_c = 10 m/s (**a**), no pathways are present, while for 1 m/s (**b**) structures corresponding to pathways appear. At 0.1 m/s (**c**), a number of local energy minima appear, that reflect a multitude of pathways present. A pathway separation becomes impossible, as recrossing events dominate (see Supplementary Figure 3).

termediate states in the nonequilibrium energy profile along the first two PCs. While the structure in the first two PCs becomes better resolved in simulations with 0.1 m/s pulling velocity, sorting trajectories into unique pathways becomes unfeasible due the dominance of recrossing events between the pathways (Supplementary Figure 3). This finding can be explained by assuming that single transitions over barriers, as we try to enforce by applying constraints, take place on a natural timescale between pico- and nanoseconds[41]. Forcing the system over such barriers on a time scale that is too long, i.e., with a too slow velocity, causes an artificial stationarity, resulting in the ligand accessing side states on top of or close to the barrier, which in equilibrium would not be accessed, and influence from protein conformational fluctuations. As a consequence, application of dcTMD to "real world" systems comes at the price of needing to identify such "goldilocks" velocities, with 1 m/s being a good rule of thumb velocity.

Langevin simulations

Langevin simulations used the integration scheme by Bussi and Parrinello[8]. Simulations were run for 1 μs of simulation time for NaCl at temperatures between 293 to 420 K, and 10 ms for the two protein-ligand systems, using temperatures 380 to 900 K for trypsin-benzamidine and 700 to 1350 K for the Hsp90-inhibitor complex. As system mass m, the reduced mass of the NaCl dimer (13.88 g/mol), the trypsin-benzamidin (120.15 g/mol) and Hsp90-inhibitor (288.73 g/mol) complexes were used. System coordinates were written out each 1 ps for NaCl and each 1 ns for the protein-ligand systems.

SUPPLEMENTARY TABLE 1. Convergence of the Bussi-Parrinello integration scheme[8] of the Langevin equation with respect to the integration time step Δt and the mass m used in the Langevin equation. The first table shows dissociation and association times obtained from 10 simulations of NaCl at T = 293 K over 1 µs simulated time, as well as results from a 1 µs long MD simulation. The other two tables show results obtained for 5 simulations of trypsin-benzamidine at T = 800 K over 0.2 ms simulated time, and 5 simulations of Hsp90-inhibitor complex at T = 1200 K over 0.2 ms simulated time, respectively. Errors denote the standard error of the mean.

**NaCl**	normal mass		mass ×10	
Δt (fs)	τ_{diss} (ps)	τ_{assoc} (ps)	τ_{diss} (ps)	τ_{assoc} (ps)
0.1	406 ± 8	3,248 ± 66	464 ± 7	3,580 ± 50
0.2	404 ± 8	3,174 ± 63	467 ± 12	3,528 ± 80
0.5	411 ± 8	3,166 ± 65	457 ± 9	3,392 ± 48
1.0	428 ± 9	3,157 ± 65	448 ± 8	3,266 ± 78
2.0	403 ± 8	3,162 ± 63	449 ± 8	3,353 ± 39
5.0	420 ± 9	3,035 ± 61	468 ± 13	3,454 ± 53
10.0	345 ± 6	2,631 ± 47	452 ± 10	3,512 ± 71
20.0	249 ± 5	1,907 ± 25	468 ± 10	3,475 ± 67
MD	124 ± 6	848 ± 47	124 ± 6	848 ± 47

**Trypsin**	normal mass		mass ×10	
Δt (fs)	τ_{diss} (ns)	τ_{assoc} (ns)	τ_{diss} (ns)	τ_{assoc} (ns)
0.5	111 ± 2	32 ± 1	112 ± 3	33 ± 1
1.0	106 ± 2	32 ± 1	111 ± 1	34 ± 1
2.0	96 ± 3	28 ± 1	111 ± 3	33 ± 1
5.0	61 ± 2	18 ± 1	112 ± 5	33 ± 1
10.0	37 ± 1	10 ± 1	105 ± 5	32 ± 1
20.0	–	–	95 ± 4	28 ± 1

**Hsp90**	normal mass		mass ×10	
Δt (fs)	τ_{diss} (ns)	τ_{assoc} (ns)	τ_{diss} (ns)	τ_{assoc} (ns)
0.5	628 ± 25	60 ± 6	641 ± 36	61 ± 5
1.0	635 ± 50	60 ± 4	638 ± 38	63 ± 6
2.0	567 ± 18	56 ± 3	636 ± 35	63 ± 4
5.0	356 ± 12	36 ± 2	621 ± 34	63 ± 6
10.0	212 ± 8	20 ± 1	608 ± 21	60 ± 5
20.0	–	–	545 ± 41	55 ± 4

For each system, we studied the convergence of the Bussi-Parrinello integrator with respect to the time step Δt, see Table 1. We find that NaCl requires an integration time step of $\Delta t \lesssim 5\,\mathrm{fs}$, while the protein-ligand systems require a significantly shorter time step of $\Delta t \lesssim 1\,\mathrm{fs}$. Owing to the Fourier relation $\Delta E \Delta t \sim \hbar$, this finding is a consequence of the larger barrier height ΔE of the free energy landscape of the protein-ligand systems.

To test if the dynamics is overdamped, Langevin simulations were repeated using a mass that is ten times larger than the normal reduced mass. (Overdamped dynamics neglects the inertia term and therefore does not depend on the mass[42].) Since the resulting (un)binding times do not change for the protein-ligand systems, these systems are clearly overdamped. By using the enhanced mass, the protein-ligand systems can therefore be integrated by using a time step of $\Delta t \lesssim 10\,\mathrm{fs}$, and therefore require an order of magnitude less simulation time. Moreover, the overdamped limit allows us to circumvent the definition of the effective mass associated with a given pulling coordinate, which depends on various technical issues such as the definition of the pulling centers. On the other hand, NaCl shows a 15 % increase of the dissociation and association times, and may be therefore classified as almost overdamped.

The gradient of the potential of mean force was approximated as

$$\frac{\mathrm{d}G(x)}{\mathrm{d}x} \approx \frac{[\Delta G(x+\Delta x) - \Delta G(x)] + [\Delta G(x) - \Delta G(x-\Delta x)]}{2\Delta x} \qquad (1)$$

Input free energy and fiction fields obtained from dcTMD were smoothed with a Gauss filter ($\sigma = 10$ for NaCl and 40 ps for protein-ligand systems, respectively). In some cases, though, friction fields still exhibited negative values after smoothing, which we found to be a consequence of not completely converged friction profiles. The problem can be circumvented by improved sampling or an increased $\sigma = 100$ ps, which we used in the preparation of Figures 3 and 4 in the main text. As workaround, we found that using the absolute values $|\Gamma(x)|$ after smoothing with $\sigma = 40$ ps as input for simulations is sufficient as well, provided that we have at least 80–100 trajectories available for a pathway of interest. This workaround was used to prepare fields for protein-ligand Langevin simulations. For x, we used a resolution of 1 pm. For compensation of data borders, we employed "fully reflective" boundary conditions: If the system jumped over a boundary x_{max} at any time step by a distance a, it was put back to $x = x_{\mathrm{max}} - a$, and its velocity sign reversed.

Mean waiting times τ were calculated by defining geometric cores[43]: For NaCl, the free energy surface was separated into the bound state $x < 0.31$ nm and unbound state $x > 0.43$. For trypsin-benzamidine, we used a bound state $x < 0.3$ nm and unbound state $x > 0.6$ nm, while for the Hsp90-inhibitor complex, we applied cores of $x < 0.3$ nm and $x > 0.9$ nm. As the native units of k_{on} are $s^{-1}\,M^{-1}$, all according binding rates were scaled by a pulling coordinate-dependent reference concentration $C = 1/\left(\frac{4}{3}\pi x_{\mathrm{ref}}^3\right)$ for one ion or protein-ligand pair with $x_{\mathrm{ref}} = x_{\mathrm{end}} - x_0$, amounting to a molarity of 0.2 M for NaCl Langevin simulations and 50 mM for protein-ligand Langevin simulations.

To demonstrate the effect of constraints used in dcTMD, Supplementary Figure 5 displays a comparison of friction fields of the NaCl-water system obtained from dcTMD and a data-driven Langevin equation (dLE)[44]. The latter uses no constraints, but calculates the friction via a local average[44]. The dLE was applied to 200 ns unbiased equilibrium MD data and the consistency of the Γ estimate was verified by comparing MD and dLE dynamics. While both

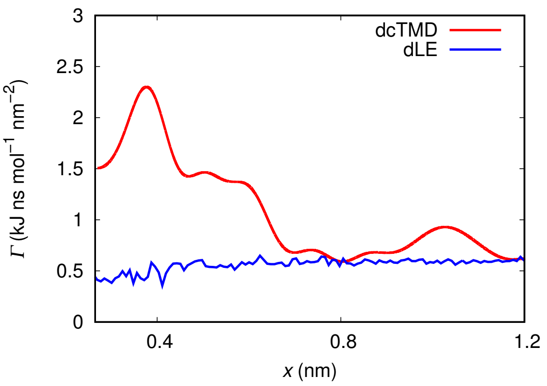

SUPPLEMENTARY FIGURE 5. **Comparison of friction estimates.** Friction calculated from a data-driven Langevin equation[44] in comparison to results from dcTMD, obtained for the example of NaCl in water.

fields differ in the details of shape, the dLE field reaches approximately the final friction value from dcTMD in the unbound state at maximal x. On average, we find that dcTMD clearly overestimates the friction, which presumably is caused by the moving position constraints[45].

Uncertainty for prediction of rates with temperature boosting

To estimate the extrapolation error of T-boosting, we assume that the waiting time τ of an unbinding or binding process is exponentially distributed,

$$P(\tau) = \frac{1}{\langle\tau\rangle} \mathrm{e}^{-\frac{\tau}{\langle\tau\rangle}}, \tag{2}$$

where $\langle\tau\rangle$ is a function of the temperature T. Hence the expectation $\bar{\tau}$ is given by its mean $\langle\tau\rangle$ plus/minus the error of the mean

$$\bar{\tau}(T) = \langle\tau(T)\rangle \pm \frac{\langle\tau(T)\rangle}{\sqrt{N(T)}}, \tag{3}$$

where $N(T)$ denotes the number of simulated transitions. By changing to the dimensionless rate $k = t_0/\langle\tau\rangle$ (with t_0 being some timescale, e.g., ns) and employing Gaussian error propagation to lowest order[46], we obtain accordingly

$$\ln\left(\bar{k}(T)\right) = \ln\left(k(T)\right) \pm \frac{1}{\sqrt{N(T)}}. \tag{4}$$

Due to the rate expression $k \propto e^{-\Delta G/k_\mathrm{B}T}$ with transition state energy ΔG, $\ln(k)$ depends linearly on $1/T$, i.e.,

$$\ln(k(T)) = \frac{a}{T} + b. \tag{5}$$

Linear regression theory[46] yields estimates for a and b as well as uncertainties

$$\sigma_b = \sqrt{\frac{\sum_i \frac{N(T_i)}{T_i^2}}{\Delta}} \tag{6}$$

and

$$\sigma_a = \sqrt{\frac{\sum_i N(T_i)}{\Delta}} \tag{7}$$

with

$$\Delta = \sum_i N(T_i) \sum_i \frac{N(T_i)}{T_i^2} - \left(\sum_i \frac{N(T_i)}{T_i} \right)^2, \tag{8}$$

where T_i denotes a discrete set of temperatures at which simulations are performed. Employing error propagation, we estimate the uncertainty of $\ln(k)$ at $T_{\text{ref}} = 300$ K as

$$\sigma_{\ln(k(T_{\text{ref}}))} = \sqrt{\left(\frac{\sigma_a}{T_{\text{ref}}} \right)^2 + \sigma_b^2}. \tag{9}$$

This yields for the desired relative uncertainty of the average waiting time

$$\bar{\tau}(T_{\text{ref}}) = \langle \tau(T_{\text{ref}}) \rangle \pm \langle \tau(T_{\text{ref}}) \rangle \cdot \sigma_{\ln(k(T_{\text{ref}}))}. \tag{10}$$

To illustrate the typical magnitude of this uncertainty, we assume that we perform 10 Langevin simulations of length t_{LE} at different temperatures T_i ($i = 0, \ldots, 9$)

$$T_i = T_0 + i \left(25 \frac{T_0}{T_{\text{ref}}} \right) \text{K}. \tag{11}$$

The first three temperatures (T_0, T_1 and T_2) are chosen such that we observe $\approx 10^2$ transitions during the simulation time t_{LE}. Similarly, we assume to observe 10^3 transitions at T_3, T_4 and T_5, 10^4 transitions at T_6, T_7 and T_8 and 10^5 transitions at T_9. Using the case of $T_0 = T_{\text{ref}} = 300$ K, since we assumed 10^2 transitions at $T_0 = T_{\text{ref}}$ during the Langevin simulation time t_{LE}, the observed rate at 300 K is $k = 10^2/t_{\text{LE}}$. Choosing $t_{\text{LE}} = 5$ ms, the corresponding rate is $k(300\text{K}) = 1/50\mu\text{s}$, and we obtain for the error of the rate $\sigma_{\ln(k(T_{\text{ref}}))} = 7.7\%$. Alternatively, when we assume that we need to choose $T_0 \approx 450$ K in order to achieve 10^2 transitions, employing the boosting relation (4) in the main text and $t_{\text{LE}} = 5$ ms, the observed rate at 300 K is $k(300\text{K}) = 0.063$ ms^{-1} with an error of 10.6%.

Considering our Langevin simulations of trypsin described in the main text, where we used T-boosting at 13 temperatures from 380–900 K, the error at 300 K is estimated to be $\sigma_{\ln(k(T_{\text{ref}}))} = 3.3\%$. For Hsp90, the system with the highest considered barrier, we obtain $\sigma_{\ln(k(T_{\text{ref}}))} = 11.0\%$ at 300 K using Langevin simulations at 14 temperatures from 700–1350 K. As the overestimation of friction factors due to usage of constraints (see main text) results in a underestimation of rates by a factor of ~ 4, and as the error of free energy profiles enters Eq. (4) in the main text in the exponent, the extrapolation error due to T-boosting can easily be made negligible in all practical cases.

SUPPLEMENTARY REFERENCES

[1]Hornak, V. *et al.* Comparison of multiple Amber force fields and development of improved protein backbone parameters. *Proteins* **65**, 712–725 (2006).

[2]Best, R. B. & Hummer, G. Optimized molecular dynamics force fields applied to the helix-coil transition of polypeptides. *J. Phys. Chem. B* **113**, 9004–9015 (2009).

[3]Jorgensen, W. L., Chandrasekhar, J., Madura, J. D., Impey, R. W. & Klein, M. Comparison of simple potential functions for simulating liquid water. *J. Chem. Phys.* **79**, 926 (1983).

[4]Abraham, M. J. *et al.* Gromacs: High performance molecular simulations through multi-level parallelism from laptops to supercomputers. *SoftwareX* **1**, 19 – 25 (2015).

[5]Olsson, M. H., Søndergaard, C. R., Rostkowski, M. & Jensen, J. H. PROPKA3: consistent treatment of internal and surface residues in empirical p K a predictions. *J. Chem. Theory Comput.* **7**, 525–537 (2011).

[6]Darden, T., York, D. & Petersen, L. Particle mesh Ewald: An N log(N) method for Ewald sums in large systems. *J. Chem. Phys.* **98**, 10089 (1993).

[7]Hess, B., Bekker, H., Berendsen, H. J. C. & Fraaije, J. G. E. M. LINCS: A linear constraint solver for molecular simulations. *J. Comp. Chem.* **18**, 1463–1472 (1997).

[8]Bussi, G., Donadio, D. & Parrinello, M. Canonical sampling through velocity rescaling. *J. Chem. Phys.* **126**, 014101 (2007).

[9]Berendsen, H. J. C., Postma, J. P. M., van Gunsteren, W. F., Dinola, A. & Haak, J. R. Molecular dynamics with coupling to an external bath. *J. Chem. Phys.* **81**, 3684 (1984).

[10]Wolf, S. & Stock, G. Targeted molecular dynamics calculations of free energy profiles using a nonequilibrium friction correction. *J. Chem. Theory Comput.* **14**, 6175–6182 (2018).

[11]Ryckaert, J. P., Ciccotti, G. & Berendsen, H. J. C. Numerical-integration of cartesian equations of motions of a system with constraints-molecular dynamics of n-alkanes. *J. Comput. Phys.* **23**, 327–341 (1977).

[12]Parrinello, M. & Rahman, A. Polymorphic transitions in single crystals: A new molecular dynamics method. *J. Appl. Phys.* **52**, 7182–7190 (1981).

[13]Wang, J. & Brüschweiler, R. 2D entropy of discrete molecular ensembles. *J. Chem. Theory Comput.* **2**, 18–24 (2006).

[14]Sousa da Silva, A. W. & Vranken, W. F. ACPYPE - AnteChamber PYthon Parser interfacE. *BMC Res. Notes* **5**, 367 (2012).

[15]Wang, J. M., Wolf, R. M., Caldwell, J. W., Kollman, P. A. & Case, D. A. Development and testing of a general amber force field. *J. Comput. Chem.* **25**, 1157–1174 (2004).

[16]Bayly, C. I., Cieplak, P., Cornell, W. D. & Kollman, P. A. A well-behaved electrostatic potential based method using charge restraints for deriving atomic charges: The resp model. *J . Phys. Chem.* **97**, 10269–10280 (1993).

[17]Neese, F. The ORCA program system. *WIREs Comput. Mol. Sci.* **2**, 73–78 (2012).

[18]Lu, T. & Chen, F. Multiwfn: A multifunctional wavefunction analyzer. *J. Comput. Chem.* **33**, 580–592 (2012).

[19]Marquart, M., Walter, J., Deisenhofer, J., Bode, W. & Huber, R. The geometry of the reactive site and of the peptide groups in trypsin, trypsinogen and its complexes with

inhibitors. *Acta Crystallogr. B* **39**, 480–490 (1983).
[20]Guillain, F. & Thusius, D. Use of proflavine as an indicator in temperature-jump studies of the binding of a competitive inhibitor to trypsin. *J. Am. Chem. Soc.* **92**, 5534–5536 (1970).
[21]Wolf, S. *et al.* Estimation of Protein-Ligand Unbinding Kinetics Using Non-Equilibrium Targeted Molecular Dynamics Simulations. *J. Chem. Inf. Model.* **59**, 5135–5147 (2019).
[22]Jakalian, A., Bush, B. L., Jack, D. B. & Bayly, C. I. Fast, efficient generation of high-quality atomic Charges. AM1-BCC model: I. Method. *J. Comput. Chem.* **21**, 132–146 (2000).
[23]Jakalian, A., Jack, D. B. & Bayly, C. I. Fast, efficient generation of high-quality atomic charges. AM1-BCC model - II. Parameterization and validation. *J. Comput. Chem.* **23**, 1623–1641 (2002).
[24]Güldenhaupt, J. *et al.* Ligand-Induced Conformational Changes in HSP90 Monitored Time Resolved and Label Free-Towards a Conformational Activity Screening for Drug Discovery. *Angew. Chem. Int. Ed.* **57**, 9955–9960 (2018).
[25]Michaud-Agrawal, N., Denning, E. J., Woolf, T. B. & Beckstein, O. Mdanalysis: A toolkit for the analysis of molecular dynamics simulations. *J. Comput. Chem.* **32**, 2319–2327 (2011).
[26]Sittel, F., Jain, A. & Stock, G. Principal component analysis of molecular dynamics: On the use of Cartesian vs. internal coordinates. *J. Chem. Phys.* **141**, 014111 (2014).
[27]Kluyver, T. *et al.* Jupyter Notebooks-a publishing format for reproducible computational workflows. In Loizides, F. & Schmidt, B. (eds.) *Positioning and Power in Academic Publishing*, 87–90 (IOP Press, 2016).
[28]van der Walt, S., Colbert, S. C. & Varoquaux, G. The NumPy Array: A Structure for Efficient Numerical Computation. *Comput. Sci. Eng.* **13**, 22 (2011).
[29]Virtanen, P. *et al.* SciPy 1.0: fundamental algorithms for scientific computing in Python. *Nat. Methods* **17**, 261–272 (2020).
[30]Price-Whelan, A. M. *et al.* The astropy project: Building an open-science project and status of the v2.0 core package. *Astron. J.* **156**, 123 (2018).
[31]Hunter, J. D. Matplotlib: A 2D graphics environment. *Comput. Sci. Eng.* **9**, 90–95 (2007).
[32]Schrödinger, LLC. The PyMOL Molecular Graphics System, Version 1.8 (2010).
[33]Efron, B. & Stein, C. The Jackknife Estimate of Variance. *Ann. Stat.* **9**, 586–596 (1981).
[34]Pearson, E. S. I. Note on Tests for Normality. *Biometrika* **22**, 423–424 (1931).
[35]Amaral, M. *et al.* Protein conformational flexibility modulates kinetics and thermodynamics of drug binding. *Nat. Commun.* **8**, 2276 (2017).
[36]Hall, R., Dixon, T. & Dickson, A. Correction Terms for Calculating Binding Free Energy Using Rates from Nonequilibrium Simulations. *Preprint at doi.org/10.26434/chemrxiv.11904915.v1* (2020).
[37]Post, M., Wolf, S. & Stock, G. Principal component analysis of nonequilibrium molecular dynamics simulations. *J. Chem. Phys.* **150**, 204110 (2019).
[38]Ernst, M., Sittel, F. & Stock, G. Contact- and distance-based principal component analysis of protein dynamics. *J. Chem. Phys.* **143**, 244114 (2015).
[39]Bray, S. *Approaches to analyzing protein-ligand dissociation with targeted molecular dy-*

namics (Master Thesis, 2018).

[40]Bryant, D. & Moulton, V. Neighbor-net: an agglomerative method for the construction of phylogenetic networks. *Mol. Biol. Evol.* **21**, 255–265 (2004).

[41]Dror, R. O. *et al.* Pathway and mechanism of drug binding to G-protein-coupled receptors. *Proc. Natl. Acad. Sci. USA* **108**, 13118–13123 (2011).

[42]Berendsen, H. J. C. *Simulating the Physical World* (Cambridge University Press, Cambridge, 2007).

[43]Nagel, D., Weber, A., Lickert, B. & Stock, G. Dynamical coring of Markov state models. *J. Chem. Phys.* **150**, 094111 (2019).

[44]Schaudinnus, N., Lickert, B., Biswas, M. & Stock, G. Global Langevin model of multidimensional biomolecular dynamics. *J. Chem. Phys.* **145**, 184114 (2016).

[45]Daldrop, J. O., Kowalik, B. G. & Netz, R. R. External potential modifies friction of molecular solutes in water. *Phys. Rev. X* **7**, 041065 (2017).

[46]Hughes, I. & Hase, T. *Measurements and Their Uncertainties.* A Practical Guide to Modern Error Analysis (Oxford University Press, 2010).
